# The effect of animacy on the agent preference: Self-paced reading evidence from Basque

**DOI:** 10.3758/s13421-025-01698-w

**Published:** 2025-04-30

**Authors:** Aitor Egurtzegi, Sebastian Sauppe, Arrate Isasi-Isasmendi, Gillen Martinez de la Hidalga, Ina Bornkessel-Schlesewsky, Matthias Schlesewsky, Itziar Laka, Martin Meyer, Balthasar Bickel, Caroline Andrews

**Affiliations:** 1 English Department, University of Zurich, Zürich, Switzerland; 2 Department of Comparative Language Science, University of Zurich, Zürich, Switzerland; 3 Center for the Interdisciplinary Study of Language Evolution, University of Zurich, Zürich, Switzerland; 4 Department of Psychology, University of Zurich, Zürich, Switzerland; 5 Department of Linguistics and Basque Studies, University of the Basque Country (UPV/EHU), Bilbao, Spain; 6 Cognitive Neuroscience Laboratory, Australian Research Centre for Interactive and Virtual Environments, University of South Australia, Adelaide, Australia

**Keywords:** Incremental argument interpretation, Agent preference, Typology, Self-paced reading, Basque, Animacy

## Abstract

Language processing shows a tendency to prefer agents over other roles. For instance, when initial unmarked noun phrases (NPs) are ambiguous between agent and patient roles, there is a preference to interpret them as agents, with ensuing reanalysis effects if the NP later turns out to be a patient. Intriguingly, this preference also applies in languages where initial, unmarked (caseless) NPs would tend to be patients because agents are often dropped or marked by a distinct case, the ergative. However, an unresolved question is to what extent the agent preference can be modulated by animacy in a language with agent-dropping and ergative case. To address this, we performed a self-paced reading study exploiting a case-marking syncretism in Basque, which makes some NPs ambiguous between agent and patient readings despite otherwise consistent ergative marking of agents. We looked at the role of an animate vs. inanimate initial NP in transitive sentences, modeling self-paced reading times in a hierarchical Bayesian regression framework. When the role of the initial NP was disambiguated by the verb, we found no reanalysis effect. By contrast, when the role of the initial NP was disambiguated by a second, unambiguous NP, we found a slowdown after human patients compared to human agents, but not after inanimate patients, in the words following the disambiguating region. This suggests that the agent preference can be attenuated when initial NPs are inanimate.

**Supplementary Information:**

The online version contains supplementary material available at 10.3758/s13421-025-01698-w.

## Introduction

Sentences are parsed incrementally: each new word is integrated into the sentence structure that the language user is building in real-time (Altmann & Steedman, 1988; Frazier & Rayner, 1982; Marslen-Wilson, 1973). This process is facilitated by various strategies. One well-established strategy is known as the agent (or subject) preference: the processing system has a preference to analyze initial arguments as agents^1^ when the role information is ambiguous. Thus, initial noun phrases (NPs) that are ambiguously marked and could be compatible with agent or patient readings tend to be interpreted as agents. For instance, in the German verb-final clause in (1a) from Haupt et al. (2008), the case marking on *Bertram* is ambiguous between the nominative, accusative, and dative case, and therefore the case does not resolve the role (nor the grammatical function) of the first NP the way that unambiguous case marking would. In fact, the role is not resolved until the parser reaches the auxiliary *hat*, which agrees with a singular agent/subject and therefore is only compatible with *Bertram* being the agent/subject and not the plural *Surferinnen* ‘surfers (female)’. Rather than remaining agnostic about the roles until the auxiliary disambiguates them, comprehenders parse it assuming *Bertram* is the agent.
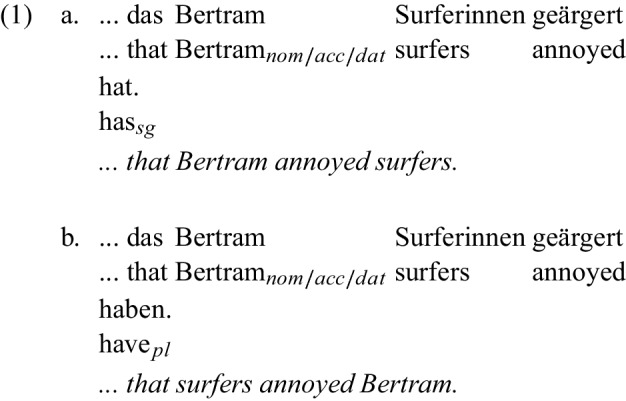


Haupt et al. (2008) demonstrated this agent preference by comparing sentences like (1a) to sentences like (1b), in which the agreement on the auxiliary is plural, forcing an interpretation in which *Surferinnen* is the agent. The disambiguation point at the auxiliary in (1b) showed an N400 relative to (1a), indicating that (1b) was reanalyzed to fit the patient-initial reading of *Bertram*. Haupt et al. ’s finding is in line with earlier work on reanalysis effects in these constructions (Bader & Meng, 1999; Bornkessel & Schlesewsky, 2006; Frazier & d’Arcais, 1989; Friederici et al., 2001; Hemforth et al., 1993; Schlesewsky et al., 2000, among others).

The agent preference has been shown to be largely robust across a number of dimensions of language variation. For instance, it persists in languages that exhibit “ergative" case: a special, non-default case reserved for the agents/subjects of transitive events (Erdocia et al., 2009; Bickel et al., 2015; Foley, 2020). Ergative case is an important test because it would bias the statistics of unmarked nouns in the language toward being patients, not agents. The agent preference is also robust in terms of the extent to which agents tend to be omitted in discourse (Bickel et al., 2015; Demiral et al., 2008; Wang et al., 2009), which can also weaken the tendency for initial NPs to be agents, making the agent preference a more risky processing strategy to employ. Similarly, it persists when the default word order of the language is OSV (i.e., patient-agent-verb), as shown by Sauppe et al. (2023) for the Austronesian language Äiwoo.

These findings make the agent preference a candidate for a universal processing strategy, at least in simple transitive clauses. However, there are still potential ways in which the agent preference could be modulated. So far, the constructions that have been tested typically gave insight into how resilient the agent preference is when the feature that varied was chiefly case grammar and usage. What has been less in focus are intrinsic semantic properties of the NP that impact how “good" an agent that NP can be, such as animacy. However, there are suggestions from outside the agent preference literature that the parser actively uses features like animacy to make decisions about agentivity. Therefore, this paper tests the robustness of the agent preference to changes in these intrinsic properties of the NP.

### Noun phrase animacy in sentence comprehension

Animacy is closely linked to the linguistic implementation of agentivity. Instead of a single unified concept, definitions of agentivity tend to describe it as a cluster of properties: no single feature is necessary, but the more relevant properties a sentence participant has, the more agentive it is (e.g., Dowty, 1991). Animacy can be viewed as one of these properties itself, or it can be related to those properties. Animate referents, humans in particular, are much more likely to have agentive properties such as volitionality and sentience (Tunmer, 1985), which inanimate referents lack. Thus, mapping animacy types to roles would prototypically correlate inanimate nouns with the patient role, and human nouns with the agent role (Comrie, 1989; Silverstein, 1976). Based on this, Bornkessel and Schlesewsky (2006) and Bornkessel-Schlesewsky and Schlesewsky (2009b) argued that, if an ambiguously marked inanimate NP was initially assigned the agent role, reassigning it to a patient should be less costly for the parser than if a human NP were to be reassigned from an agent to a patient role.

Consistent with this, animacy has been found to be an active feature in parsing. The reassignment of an ambiguous second NP that turns out to be an agent is faster when the first NP is inanimate, as research on English has revealed (Clifton, 1992, 1993). Animacy was also found to influence processing in Zurich German and Fering, where patient-initial sentences were rated faster when the patient was inanimate and the agent was human, compared to when both roles were expressed by human referents (Dröge et al., 2020). Further, Kretzschmar et al. (2012) found that while there was an additional processing cost associated with patient-initial sentences in German, verbs that require dative arguments had a smaller difference between agent-initial and patient-initial sentences, since the NPs in these constructions are less agentive and thus also less prototypical agents (cf. also Bornkessel et al., 2004).

However, the effect of animacy is not uniform across the literature. Working with locally ambiguous *Wh-* questions in German, Schlesewsky et al. found an agent preference for both human and inanimate NPs (Schlesewsky et al., 2000). Similarly, Turkish comprehenders showed a preference for agents in initial position, with no ERP differences between human and inanimate agents (Demiral et al., 2008). For Mandarin Chinese, an N400 ERP effect was found for patient-initial structures when compared to agent-initial structures that had either inanimate or human agents (Wang et al., 2009).

Apart from research on main clauses, there has been work on relative clause comprehension, which includes a matrix clause and an embedded clause, and thus adds another layer of complexity. Evidence from relative clauses has shown that while patient (object) relative clauses are generally harder to parse than agent (subject) relative clauses, the comprehension of patient relative clauses depends on the animacy of the initial NP. For English and Spanish, patient relative clauses showed a faster reanalysis of inanimates as patients compared to human NPs (Traxler et al., 2005; Betancort et al., 2009). For Dutch, it has been found that agent-relative clauses are read faster than patient-relative clauses also when the agent is inanimate (Mak et al., 2006).

Moreover, grammaticality violation effects in agents are stronger for human than inanimate agent relative clauses in Hebrew (Deutsch & Bentin, 2001), and integrating inanimate agents is costlier than integrating human agents in English (Gennari & MacDonald, 2008; Lowder & Gordon, 2012).

Notably, none of the languages that have previously been investigated for how animacy affects the agent preference have an ergative case marking system. The availability of animacy effects elsewhere in sentence comprehension, combined with some particular features of ergative case alignment, means that it is worth investigating ergative languages, independently of the findings from languages with non-ergative case alignments.

### Ergativity in sentence comprehension

Research on animacy effects has so far been limited primarily to languages where agents are unmarked and patients are marked (by accusative or dative case, or word order), i.e., where case-marking follows a nominative-accusative pattern. Ergative case systems, however, pose an additional challenge to the agent preference: Because agents are marked by special ergative case morphology, unmarked noun phrases could in principle default to a patient reading. Even though this strongly disfavors the agent preference, its effects were still found in Hindi for inanimate NPs. Bickel et al. (2015) found a N400 difference for disambiguations of role-ambiguous NPs to patient readings compared to agent readings, despite the fact that inanimate NPs tend to be patients in language use.

However, the evidence from other languages with ergative case marking is more contradictory. For example, in Georgian, Foley (2020) found a preference for ergative-case marked sentences at the verb for human NPs, though nothing with nominative marking. However, in a separate study with only inanimate initial NPs, they found a patient preference at the verb for nominative-aligned sentences, indicating that patient preferences are possible with inanimates, in contrast to Bickel et al. (2015). In Avar (Northeast Caucasian), on the other hand, no differences were found between ergative agents and absolutive patients in relative clauses (Polinsky et al., 2012). However, this study did not contrast animate and inanimate noun phrases. Meanwhile, Bickel et al. (2015) only looked at inanimates, and Foley ’s studies did not compare animates and inanimates within the same study.

One issue that has hampered progress is that the evidence for animacy effects under ergative case has been limited to languages where ergative case is assigned to agents only under specific conditions. In Hindi, the ergative is assigned only to agents of transitive sentences in the perfective aspect (Mahajan, 1990); similarly, in Georgian the ergative is assigned by transitive verbs and by some (active) intransitive verbs, in specific tense-aspect-mood combinations (Harris, 1990). In response to this, we turn to a language that assigns ergative case consistently to all agents and leaves unmarked all patients (‘absolutive’ or ‘nominative’ case). This is Basque, a language with verb-final default order and frequent agent dropping. Most importantly, Basque deviates from the classic cross-linguistic ergative pattern because the ergative appears on the arguments of agentive intransitive verbs as well. This means that the correspondence between the ergative case and the agentive semantic role is much higher than in other languages. The assignment of ergative case to agents in Basque can be seen in the following transitive and intransitive sentences, both in the perfective and the imperfective aspects.
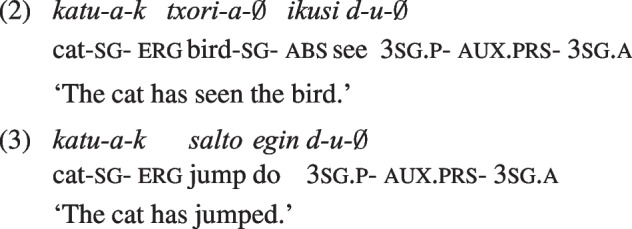

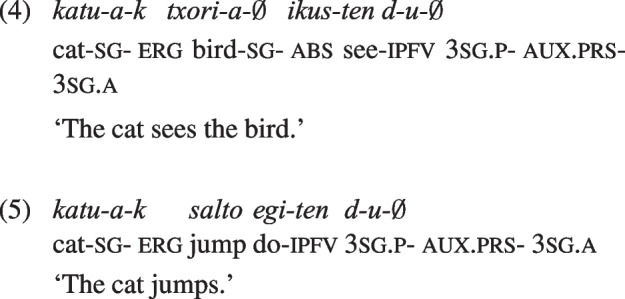


Erdocia et al. (2009) investigated the agent preference in Basque by presenting participants with NP-NP-VP sentences with agent-patient-verb (APV) or patient-agent-verb (PAV) word order. All nouns were animate, comprising both humans and animals, and presented semantically coherent scenarios (such as “the wolf ate the sheep”). Crucially, their manipulation took advantage of a syncretism in the Basque case paradigm that gives rise to an ambiguity between agent and patient marking: ergative singular and absolutive plural are realized with the same phonological form (see Table 1).

**Table 1:** Part of the case marking paradigm in Basque, with absolutive and ergative case morphemes for singular and plural nouns, adapted from Manterola (2008)

	Absolutive	Ergative
Singular	-a-$\emptyset$	-a-k
	sg-abs	sg-erg
Plural	-ak-$\emptyset$	-ek
	pl-abs	pl.erg

Agents are marked by ergative case, patients by absolutive (unmarked) case

Erdocia et al. report three experiments. The first two experiments made use of the self-paced reading methodology where participants had to read both word order types. In the first experiment, NPs were always singular, where singular agents could be interpreted as plural patients (as in Table 1), but singular patients were unambiguously marked. Speakers preferred APV over PAV word order. In the second experiment, all NPs from Experiment 1 were plural, and therefore agents now were unequivocally case-marked as agents, but plural patients could be interpreted as singular agents. Additionally, they also included fully ambiguous sentences, where the NP could be interpreted as an agent or a patient (see Table 1). These sentences, however, were disambiguated through semantic world knowledge (as in the example above “the wolf ate the sheep”, not “the sheep ate the wolf”). Again, Erdocia et al. found that APV sentences were read faster than PAV sentences, even when they were fully ambiguous. In their final experiment, they looked at the ERP signal during sentence processing, finding a negativity for PAV sentences compared to APV sentences.

However, to the best of our knowledge, no work in Basque has looked at whether the agent preference also holds with inanimate nouns, which inherently make for less prototypical agents. On the one hand, the fact that the agent preference has previously been found in Basque (Erdocia et al., 2009; Yetano et al., 2019), and that it has also been found with inanimate nouns in other typologically diverse languages (Haupt et al., 2008; Demiral et al., 2008; Wang et al., 2009), including languages with variations on ergative case marking (Choudhary, 2011; Bickel et al., 2015), indicates that there could be an agent preference that also holds for inanimate nouns in Basque. On the other hand, agent interpretations have been found to be equally or less preferred to patient interpretations of ambiguous NPs, at least for relative clauses of languages with ergatives (Carreiras et al., 2010; Polinsky et al., 2012). Foley (2020) even found a patient preference for role-ambiguous inanimate NPs in Georgian.

Thus, the consistent ergative case marking for agents in Basque (Laka, 2006, 2017) does not clarify whether inanimate nouns are preferentially interpreted as patients. Here, we fill the gap left by previous research on Basque (Erdocia et al., 2009, *i.a.*), namely that of how inanimates can affect the processing of agent-initial sentences. We also control for additional factors that previous research did not consider, such as verb semantics and wrap-up effects that may arise when the disambiguating region is the final word in a sentence (Payne & Stine-Morrow, 2014).

### Hypotheses

We compared two options regarding the influence of animacy on the interpretation of role-ambiguous NPs in Basque.

First, if the agent preference is independent of animacy, ambiguous inanimate NPs should also be preferentially interpreted as agents, even if they are less prototypical agents. Second, if instead the agent preference *is* affected by animacy, there should be a difference in the interpretation of inanimate and human NPs. Disambiguations towards agent readings should be more preferred for NPs with human reference than for NPs referring to inanimates. At the same time, patient disambiguations should be preferred (or at least not penalized as strongly) for inanimate NPs, in line with their inherent properties that make them more prototypical patients than agents.

**Table 2:** Human unambiguous agent condition from a sample item from Experiment 1 (see also Table 3)

	

The disambiguating region is highlighted in gray. The adjunct and “digging-in" phrase do not change across conditions within items

We tested these comprehension preferences with reaction times in two self-paced reading experiments. In Experiment 1, the role of an ambiguous initial NP is formally disambiguated by the number agreement in the auxiliary at the end of the sentence-initial subordinate clause. In Experiment 2, the role of the initial NP is disambiguated by the unambiguous case marking of a second preverbal NP.

## Experiment 1

Experiment 1 leveraged the ambiguities created by the overlap between the absolutive plural case marker and the singular ergative marker (as in Table 1). Given that these two case-number combinations have the same form, a word ending in*-ak* is ambiguous: it can be interpreted as either a singular agent (ergative) or plural patient (absolutive), in either a transitive or intransitive sentence (due to the split in the intransitive system in Basque), leading to multiple possibilities for (re)interpretation.

Following the design of Bickel et al. (2015) in Hindi, we constructed transitive sentences with an NP-VP structure, dropping one of the NPs.^2^ The semantic role and animacy of the NP were manipulated across conditions (see Table 3). An example stimulus sentence is given in Table 2.

The design contrasted the animacy (human vs. inanimate), role (agent vs. patient), and ambiguity (ambiguous vs. unambiguous case marking) of the NP, as well as their interactions ($2\times 2\times 2$ design). The disambiguating region was the auxiliary verb because it carries person and number agreement that corresponds to both the agent and the patient.^3^ The complete set of conditions for this experiment can be seen in Table 3 (except for the parts that remained constant across all conditions, which can be seen in Table 2).

**Table 3:** Overview of experimental conditions for Experiment 1, showing the differences between conditions in NP case marking, lexical verb, and auxiliary verb morphology

**NP1 Human Unambiguous Agent**	Verb	AUX
Ehiztari-ek	zauritu	d-u-te-lako...
Hunter-erg.pl	wound	3.p-aux-3pl.a-comp...
Because the hunters wounded (the deer)...		
**NP1 Inanimate Unambiguous Agent**		
Bal-ek	zauritu	d-u-te-lako...
Bullet-erg.pl	wound	3.p-aux-3pl.a-comp...
Because the bullets wounded (the deer)...		
**NP1 Human Ambiguous Agent**		
Ehiztari-a-k	zauritu	d-u-e-lako...
Hunter-sg-erg	wound	3.p-aux-3sg.a-comp...
Because the hunter wounded (the deer)...		
**NP1 Inanimate Ambiguous Agent**		
Bal-a-k	zauritu	d-u-e-lako...
Bullet-sg-erg	wound	3.p-aux-3sg.a-comp...
Because the bullet wounded (the deer)...		
**NP1 Human Unambiguous Patient**		
Ehiztari-a	saihestu	d-u-te-lako...
Hunter-abs.sg	dodge	3.p-aux-3pl.a-comp...
Because (the deer) dodged the hunter...		
**NP1 Inanimate Unambiguous Patient**		
Bal-a	saihestu	d-u-te-lako...
Bullet-abs.sg	dodge	3.p-aux-3pl.a-comp...
Because (the deer) dodged the bullet...		
**NP1 Ambiguous Human Patient**		
Ehiztari-ak	saihestu	d-it-u-zte-lako...
Hunter-abs.pl	dodge	3.p-pl-aux-3pl.a-comp...
Because (the deer) dodged the hunters...		
**NP1 Inanimate Ambiguous Patient**		
Bal-ak	saihestu	d-it-u-zte-lako...
Bullet-abs.pl	dodge	3.p-pl-aux-3pl.a-comp...
Because (the deer) dodged the bullets...		

A full example stimulus sentence for one condition as presented to participants (including adjuncts and the main clause following the auxiliary) can be seen in Table 2

Participants read sentences in AV or PV word order. The sentences also included two adjuncts: One before NP1 so that they would not start with the manipulated, and another one between NP1 and the first verb, as a digging-in phrase (Paape & Vasishth, 2016) to distance NP1 from the disambiguating region, and therefore potentially increasing the size of the reanalysis effect. Additionally, the dropped NP was added back after the verb in a second clause, so that within the sentence, comprehenders had all the information they needed to resolve the event. This was intended to reduce the reliance on the discourse context to license argument-drop. Therefore, the complete sentence structure that the participants saw for each condition was Adjunct, NP1, Adjunct, VP, NP2, VP (see Table 2). In this structure, the first clause (the critical one) is a subordinate clause, which is followed by a main clause that is held constant across all conditions. Along with providing contextual support for argument-drop, the main clause allowed this design to avoid/reduce concerns about wrap-up effects on the disambiguating region (Payne & Stine-Morrow, 2014).

### Participants

Sixty-one native speakers of Basque (mean age = 33.6 years, SD = 11.2 years) participated in the experiment and were offered monetary compensation. Participants were recruited through Twitter, word of mouth, the Basque Radio Television (EITB) program “Faktoria”, and email. Ethical approval was obtained from the University of the Basque Country (M10/2017/014MR1/V1) and the University of Zürich (19.8.11).

### Materials and procedure

Forty-eight stimulus items, each with eight conditions following the pattern shown in Table 3, as well as 108 filler sentences were created. Eight different lists were created so that each participant only saw one condition of each item. The order of the sentences was randomized for each participant. A comprehension question with two possible answers to choose from was presented for half of the trials (across both critical items and fillers).

The experimental sessions started with a demographic and language background questionnaire, including questions on Basque language usage and proficiency.^4^ The participants then completed four practice trials to get used to the self-paced reading technique. Two of the practice trials were followed by a comprehension question; unique to the practice items, participants received feedback on whether they answered the question incorrectly before proceeding with the experiment. In total, the experimental sessions lasted approximately 25 minutes. The experiment was built with Ibex Farm (Drummond, 2020) and data were collected online.

### Data preprocessing

Data were preprocessed and analyzed with R (R Core Team, 2023). Nine participants were excluded from the analysis because they reported that they did not have at least one parent with whom they spoke Basque or because they reported that Basque was not their dominant language. Trials with reading times faster than 75 ms or slower than 1500 ms for at least one word were rejected (7.27% and 6.77% of all trials, respectively). Next, trials in which the reading times for at least one word were faster or slower than the average reading time ±3.5 standard deviations, calculated individually for each participant, were rejected (6.98% of all trials). Participants with more than 50% rejected trials were excluded from the analysis (six participants, 0.91% of all trials). Due to a typographical error, one condition in three different items displayed an additional word, so that the corresponding trials were excluded from analyses for these items (0.12% of trials).

**Fig. 1:**
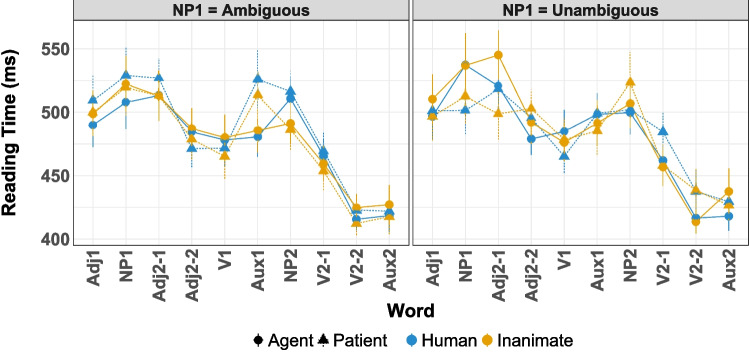
Mean reading times in Experiment 1 (based on by-participant means). Error bars indicate one standard error of the mean. The critical region was the auxiliary at the end of the initial subordinate clause (“Aux1”)

Log-transformed reading times were modeled with Bayesian hierarchical Gaussian regression, separately for each word, with the brms (Bürkner et al., 2017; Bürkner, 2017) interface to Stan (Carpenter et al., 2017). The critical predictor variables were the animacy, role, and ambiguity of the first noun phrase, as well as their interactions. Normalized word length in letters (*z*-transformed) and trial position within the experiment (also *z*-transformed) served as nuisance predictors (Sassenhagen & Alday, 2016; Schielzeth, 2010).

Random slopes for the interaction between Animacy, Role, and Ambiguity, and intercepts were added by-participants and by-items. All models used Student-*t* distributed priors (df = 5, $\mu$ = 0, $\sigma$ = 2) for all population-level predictors and half Student-*t* priors ($df=3$, $\mu$ = 0) for the random effects. The models consisted of eight chains with 8000 iterations each (including 4000 warm-up iterations). In the results figures we indicate the posterior probability of effects different from zero if the probability is at least 0.90.^5^

### Results

**Fig. 2:**
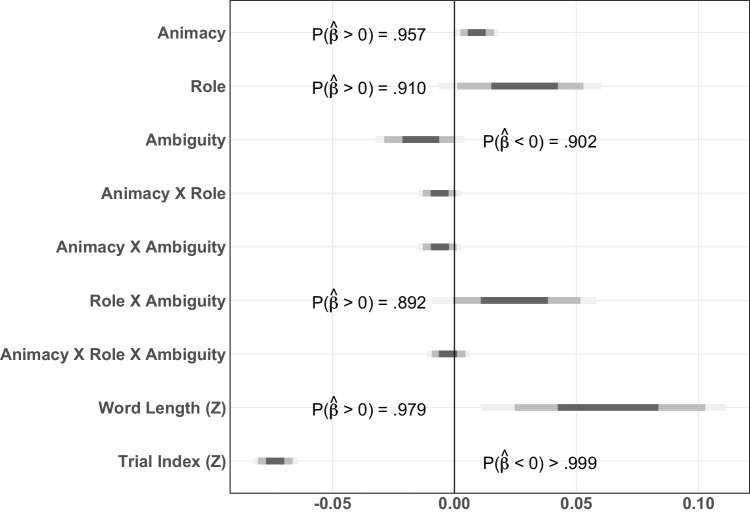
Posterior estimates for all predictors at the critical word (auxiliary verb, “Aux1") in Experiment 1

Mean reading times for Experiment 1 can be seen in Fig. 1. Posterior probability densities for the critical predictors are shown in Fig. 2 and a detailed model report can be found in Table S1 in the Supplementary Information.

The posterior probabilities suggest main effects of Animacy, Role, Ambiguity, and Word Length (Fig. 2). Counter to the predictions of either hypothesis, when the initial NP was inanimate, the auxiliary verb was read slightly faster than when the NP was a human (median $\hat{\beta }_{Animacy}$ = 0.0092, $P(\hat{\beta }>0)=0.957$, Figure 2). Additionally, when the NP was a patient, the auxiliary verb was read faster than when it referred to an agent (median $\hat{\beta }_{Role}$ = 0.0271, $P(\hat{\beta }>0)=0.910$, Figure 2). More in line with expectations, when the NP was ambiguous, the auxiliary verb was read faster (median $\hat{\beta }_{Ambiguity}$ = $-0.0143$, $P(\hat{\beta }<0)=0.902$, Figure 2).

Importantly, however, there was also a main effect of Word Length (median $\hat{\beta }_{Word\ Length}$ = 0.0632, $P(\hat{\beta }>0)=0.979$). A complication in this particular design is that word length in the disambiguating region was collinear with the role predictor. The auxiliaries in the patient-first conditions had more characters than in the agent-first conditions (*dutelako* and *duelako* compared to *dutelako* and *dituztelako*, respectively; mean auxiliary length for agent conditions = 7.31 characters, SD = 0.64 characters; mean auxiliary length for patient conditions = 9.33 characters, SD = 1.57 characters). Thus, an effect of word length could drive the semantic role-based differences in the auxiliary region (Fig. 1).

The impact of the word length difference does not stop with the main effect. The predictors Role, Ambiguity, Role $\times$ Ambiguity, and Word Length also contributed to high collinearity in this model, indicated by variance inflation factors (VIFs, see e.g., Zuur et al., 2010) greater than 3 (Role VIF $= 14.5$; Ambiguity VIF $= 4.38$; Role $\times$ Ambiguity VIF $= 14.52$; Word Length VIF $= 31.88$, see Table S1). This raises the possibility that the estimates of all of these factors are distorted by the word length confound.

**Fig. 3:**
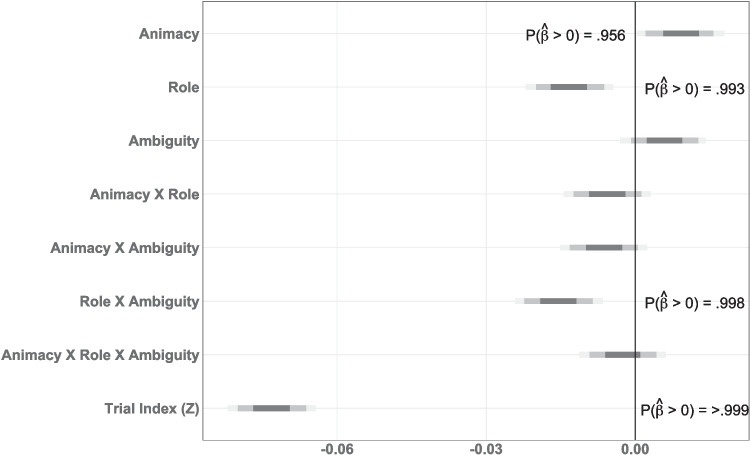
Posterior probabilities of effects at the critical word (auxiliary verb 1) in Experiment 1 (without word length as a predictor)

To assess the impact of word length on the effects in the experiment, we also fit a model without word length as a predictor (see Fig. 3 and Table S2). In this model, the directionality of most of the reliable effects switched. Without Word Length, role shows an advantage for initial agents over patients (median $\hat{\beta }_{Role}$ = $-0.0131$, $P(\hat{\beta }<0)=0.993$). The interaction of Role $\times$ Ambiguity likewise reverses (median $\hat{\beta }_{Role}$ = $-0.0058$, $P(\hat{\beta }<0)=0.998$). Finally, the effect of Ambiguity drops below the statistical threshold of credibility, but notably also trends in the opposite direction from the model with Word Length (median $\hat{\beta }_{Role}$ = $-0.0059$, $P(\hat{\beta }<0)=0.868$). This indicates that the effects from the initial model are unstable and dependent on the influence of word length for interpretation. Furthermore, a model comparison through leave-one-out cross-validation (LOO; approximated by importance sampling from the posterior) suggests that both models fit similarly well ($\Delta _{eldp} = 0.6$, SE $= 1.3$). Given the solid pre-existing theoretical motivation for including Word Length as a predictor of self-paced reading times (Ferreira & Clifton Jr, 1986; Trueswell et al., 1994, *interalia*), the model with word length is likely the better model of reference out of the two. However, this is not the same as indicating that either model is, in fact, a reliable model overall. This state of affairs, where neither model is definitively better than the other but each finds effects in the opposite direction, makes the interpretation of these results inconclusive.

### Discussion

Experiment 1 tested whether the agent preference can be modulated by animacy in transitive sentences with argument-drop. Because Basque allows both agent and patient argument-drop, we exploited this grammatical feature to produce AV and PV structures, which have both been shown to be highly frequent in sentence production (Egurtzegi, 2023). The NP VP structure in this experiment also meant that the auxiliary verb signaled the transitivity of the sentence and thus disambiguated the role of the initial NP.

The results of this experiment showed that disambiguating to an agent vs a patient led to differences in processing, but further inspection of the main effects of the model suggested that the effect is most likely attributable to (or at least significantly complicated by) Word Length. Even though the effects of NP Role, Animacy, and Ambiguity surpass a 90% credible interval threshold, given their collinearity with Word Length and the change of direction when Word Length is included, the most interpretable effect we find is a trivial effect of Word Length. However, it is possible that the collinearity between variables masked a weaker effect of Role that is not detectable in this design. Model comparison found that a model with word length as a predictor had marginally better predictive performance. More importantly, including Word Length has strong theoretical backing, so there is a good argument for taking this as the model of reference. Interestingly, however, this model showed an unusual patient preference, rather than a more expected agent preference. To the extent that this model is interpretable, the patient effect is statistically adjusted for the word length confound, and therefore there is a need to determine whether there could be construction-specific reasons for such a patient preference.

Drawing on data from an interactive description task, Egurtzegi (2023) shows that Basque speakers have a strong preference to omit (human) agents over any other role, so that agents are overtly realized only approximately 5% of the time.^6^ With this in mind, it could be the case that for the argument-drop construction in the current experiment, participants did indeed have a preference for interpreting sentences as patient-initial (manifesting in faster reading times for patient-verb structures). Readers discover that an argument was dropped when they encounter the main verb, before they reach the critical auxiliary region, and therefore could have had time to adjust their expectations, especially at the end of a clause where wrap-up integration will be taking place.^7^ Nevertheless, given the inconclusive results of the model comparison, further research should address a potential patient preference in certain Basque constructions. To resolve the problem with the collinearity between role and word length, a second experiment employed a design that minimized the word length confound at the critical region by using lexically realized agent and patient arguments. A similar design was used by Erdocia et al. (2009), who found an agent preference for human referents.

**Table 4:** Human Unambiguous Agent condition from a sample item from Experiment 2

	

The critical disambiguating region is highlighted by the gray shade. The adjunct and “digging-in" phrase do not change between conditions for a single item

**Table 5:** Overview of experimental conditions for Experiment 2, showing the differences between conditions in agent and patient case marking, lexical verb, and auxiliary verb morphology

**NP1 Human Unambiguous Agent**	**NP2 Unambiguous Patient**			Verb	AUX
Ehiztari-ek	orein-a	eta	oreinkume-a	zauritu	z-it-u-zte-n
Hunter-erg.pl	deer-abs.sg	and	fawn-abs.sg	wound	3.p.pst-pl-aux-3pl.a-pst
The hunters wounded the deer and the fawn					
**NP1 Inanimate Unambiguous Agent**	**NP2 Unambiguous Patient**				
Bal-ek	orein-a	eta	oreinkume-a	zauritu	z-it-u-zte-n
Bullet-erg.pl	deer-abs.sg	and	fawn-abs.sg	wound	3.p.pst-pl-aux-3pl.a-pst
The bullets wounded the deer and the fawn					
**NP1 Human Ambiguous Agent**	**NP2 Unambiguous Patient**				
Ehiztari-a-k	orein-a	eta	oreinkume-a	zauritu	z-it-u-e-n
Hunter-sg-erg	deer-abs.sg	and	fawn-abs.sg	wound	3.p.pst-pl-aux-3sg.a-pst
The hunter wounded the deer and the fawn					
**NP1 Inanimate Ambiguous Agent**	**NP2 Unambiguous Patient**				
Bal-a-k	orein-a	eta	oreinkume-a	zauritu	z-it-u-e-n
Bullet-sg-erg	deer-abs.sg	and	fawn-abs.sg	wound	3.p.pst-pl-aux-3sg.a-pst
The bullet wounded the deer and the fawn					
**NP1 Human Unambiguous Patient**	**NP2 Unambiguous Agent**				
Ehiztari-a	orein-ek	eta	oreinkume-ek	saihestu	z-u-te-n
Hunter-abs.sg	deer-erg.pl	and	fawn-erg.pl	dodge	3.p.pst-aux-3pl.a-pst
The deer and the fawn dodged the hunter					
**NP1 Inanimate Unambiguous Patient**	**NP2 Unambiguous Agent**				
Bal-a	orein-ek	eta	oreinkume-ek	saihestu	z-u-te-n
Bullet-abs.sg	deer-erg.pl	and	fawn-erg.pl	dodge	3.p.pst-aux-3pl.a-pst
The deer and the fawn dodged the bullet					
**NP1 Human Ambiguous Patient**	**NP2 Unambiguous Agent**				
Ehiztari-ak	orein-ek	eta	oreinkume-ek	saihestu	z-it-u-zte-n
Hunter-abs.pl	deer-erg.pl	and	fawn-erg.pl	dodge	3.p.pst-pl-aux-3pl.a-pst
The deer and the fawn dodged the hunters					
**NP1 Inanimate Ambiguous Patient**	**NP2 Unambiguous Agent**				
Bal-ak	orein-ek	eta	oreinkume-ek	saihestu	z-it-u-zte-n
Bullet-abs.pl	deer-erg.pl	and	fawn-erg.pl	dodge	3.p.pst-pl-aux-3pl.a-pst
The deer and the fawn dodged the bullets					

Elements which did not vary across conditions are excluded for space. A full condition that participants saw can be seen in Table 4, where the adjuncts and coordinated NP2 are also shown

## Experiment 2

For Experiment 2, all sentences followed a NP1-NP2-Verb structure. As in Experiment 1, the locus of the experimental manipulation was at NP1, contrasting its animacy, role, and the ambiguity of case marking. The three variables were again fully crossed with one another, creating a $2\times 2\times 2$ design. The primary difference from Experiment 1 was that the disambiguating region was NP2, which had an unambiguous case marker (either*-a* indicating NP2 was a patient or*-ek* for an agent reading of NP2). A sample of a full sentence is shown in Tables 4 and 5 shows examples of the critical, variable parts of the sentence for all conditions.

The second major difference from Experiment 1 was the addition of a noun that was coordinated with NP2 by a conjunction (NP2b coordinate in Table 2). Both of these nouns carry the same case marker, disambiguating the whole NP2 (*-a* or*-ek*, see Table 4). We will refer to these as NP2a and NP2b, respectively, and reserve NP2 for the entire coordinated NP (the words highlighted as the disambiguating region in Table 4). The additional noun ensures that spillover effects do not occur in the following verb region, but instead fall on words that are still part of the NP2 structure and identical in all conditions (see discussion in Jegerski, 2013). The critical regions where an reanalysis effect might appear are therefore the disambiguating region at NP2a, the conjunction itself (although it is quite short), and the second noun of the coordinated phrase.

As in Experiment 1, the NP1-NP2-Verb structure of the items was extended to include two adjuncts, one before NP1 so that participants would not start the sentence by reading the NP that is manipulated across conditions, and another adjunct between NP1 and NP2, as a digging-in phrase to distance the parsing of NP1 and that of NP2.

### Participants

Sixty-one participants (mean age = 33.5 years, SD = 12.2 years) participated in the experiment and were offered monetary compensation. Again, participants were recruited through Twitter, word-of-mouth, the Basque Radio Television (EITB) program “Faktoria”, and through email. Experiment 2 was conducted at the same time as Experiment 1; interested participants were given a link to an information page. If they chose to continue, they clicked a link that randomly distributed them into one of the two studies. This ensured that the populations in the two experiments were comparable. Ethical approval was obtained from the University of the Basque Country (M10/2017/014MR1/V1) and the University of Zürich (19.8.11).

### Materials and procedure

The number of stimuli, conditions, fillers, and experimental setup was the same as in Experiment 1. The filler sentences were the same as in Experiment 1.

### Data pre-processing

Data were pre-processed with R (R Core Team, 2023). Nine participants were excluded from the analysis because they reported that they did not have at least one parent with whom they spoke Basque or because they reported that Basque was not their dominant language. Three participants who had less than 80% accuracy in the comprehension questions in the experiment were excluded. Trials with reading times faster than 75 ms for a word in the trial were rejected (1.76% of trials were excluded this way), and trials where the reading time for a word was slower than 1500 ms were rejected, too (6.4% of trials). All trials with at least one word 3.5 SD away from the average reading time of each participant were rejected (6.51% of trials). Due to a typographical error, one condition in three different items displayed an additional word and was thus excluded from analyses for these items (0.06%).

The model setup was the same as in Experiment 1.

**Fig. 4:**
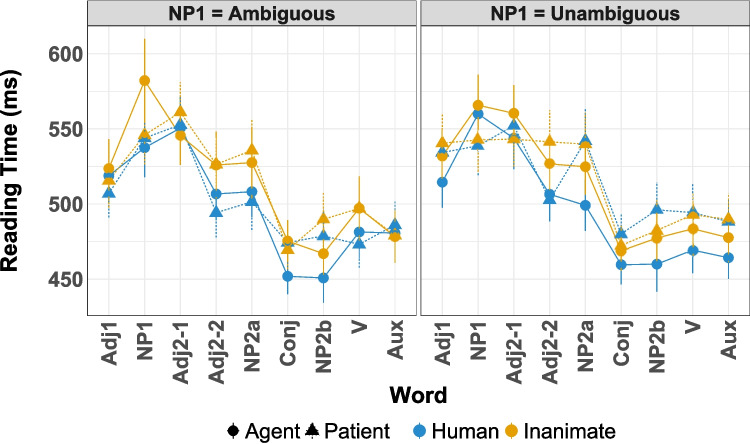
Mean reading times in Experiment 2 (based on by-participant means). *Error bars* indicate one standard error of the mean. The critical region encompasses the second noun phrase (“NP2a”, “Conj”, and “NP2b”)

**Fig. 5:**
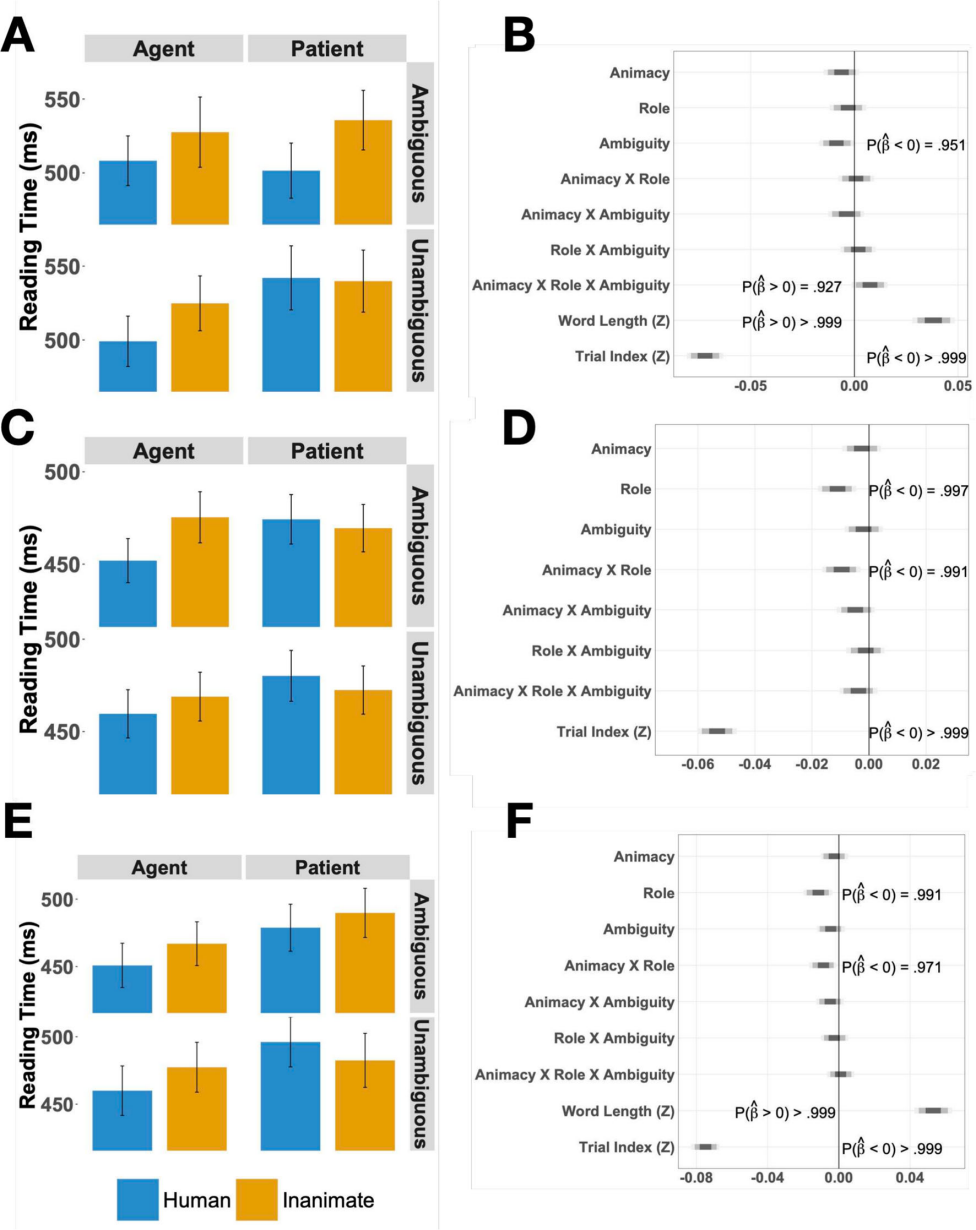
**A**: Mean reading times for critical word (NP2a) in Experiment 2; *error bars* indicate one standard error of the mean. **B**: Posterior distributions by main effects of animacy, role, ambiguity, and their interactions for critical word (NP2a) in Experiment 2. **C**: Mean reading times for critical word +1 (the conjunction) in Experiment 2; error bars indicate one standard error of the mean. **D**: Posterior distributions by main effects of animacy, role, ambiguity, and their interactions for critical word +1 (Conjunction) in Experiment 2. **E**: Mean reading times for critical word +2 (NP2b) in Experiment 2; error bars indicate one standard error of the mean. **F**: Posterior distributions by main effects of animacy, role, ambiguity, and their interactions for critical word +2 (NP2b) in Experiment 2

### Results

The mean reading times for Experiment 2 are shown in Fig. 4. Additionally, a detailed view of mean reading times for critical words and their corresponding posterior distribution plots can be found in Fig. 5. Detailed output for the models can be found in Tables S3-S7 in the Supplementary Information.

As with Experiment 1, we fit one model with all relevant predictors, including word length. For the critical word (NP2a), mean reaction times and the estimated effects are shown in Fig. 5A-B and Table S3. Given the three-way interaction (median $\hat{\beta }_{Animacy \times Role \times Ambiguity}=0.008, p(\hat{\beta }<0) = 0.927$), we calculated posterior marginal means using the emmeans package in R to better resolve the interpretation (Lenth, 2023). In emmeans, marginal means are calculated by first predicting the values for each cell in a reference grid of model variables (i.e., each condition) and using the estimates from the computed model. The marginal means themselves are then the mean of each column or row in the reference grid, for example the mean of all the estimates which are in the ’human’-level of the Animacy variable. Further subdividing which estimates are included in the mean makes it possible to calculate contrasts, for example, *patient – agent* for ambiguous referents only. Since the critical contrast is the effect of *patient – agent*, we computed it across all four combinations of animacy and semantic role.

For NP2a, the marginal means are given in Table 6. At this sentence position, the 95% HPD for the marginal means for the core agent preference contrast, ambiguous-patient minus ambiguous-agent, contains 0 for both animates and inanimates. However, to better resolve the three-way interaction, we calculated marginal means for unambiguous - ambiguous comparison. This contrast found that human unambiguous patients are read slower than human ambiguous patients, a secondary indicator contrast of the agent preference (median $\Delta \hat{\beta }{_\text {unambiguous - ambiguous}} = 0.043$, $\text {HPD}_\Delta = [0.0009, 0.0839]$). The corresponding contrast for inanimate patients shows no such ambiguity effect (median $\Delta \hat{\beta }{_\text {unambiguous - ambiguous}} = -0.0027$, $\text {HPD}_\Delta = [-0.0425, 0.0390]$).

The exact interpretation at NP2a is slightly complicated by the fact this is also the region that disambiguates away from an intransitive or argument-drop reading for the "unambiguous" patient conditions. An intransitive or agent-drop parse would have avoided an OSV reading of the unambiguous patient conditions and therefore would have been a reasonable analysis for participants to pursue prior to NP2a in this condition. Criticially, however, this only impacts human conditions and not the inanimate ones, indicating that animacy remains at the core of the parser’s decision-making at this point.

The mean reading times and the estimated effects for the conjunction after the disambiguating NP (Conj; critical word +1) are shown in Fig. 5C-D and in Table S5. A two-way interaction of Animacy $\times$ Role showed that human agents are parsed faster than any other condition, with human patients being the slowest and the inanimate conditions being similar to each other (median $\hat{\beta }_{Animacy \times Role}=-0.010, P(\hat{\beta }<0)=0.991$), again consistent with the agent preference modulated by animacy. The marginal means in Table 7 reflect this by showing longer RTs for patients than for agents in the (ambiguous) human conditions (median $\Delta \hat{\beta }_{\text {patient - agent}}= 0.0522$, $95\%\ \text {HPD}_\Delta = [0.0208, 0.0843]$, but not in the inanimate conditions. Interestingly, the marginal means and overall interactions are split on the effect of Ambiguity: the marginal means appear to indicate that semantic role has a reliable impact on processing for ambiguous human nouns but not unambiguous humans nouns, while the interactions do not indicate any role for Ambiguity. This could be a result of the ambiguous conditions catching-up to the processing difference that the unambiguous conditions showed in NP2a or an indication that the Ambiguity variable has a lesser impact at this juncture, but with the presence of the critical contrast of ambiguous-patients and ambiguous agents, either of these options is consistent with the central pattern of this data, that the agent preference modulated by animacy.

**Table 6:** 95% HPD for the marginal means at NP2a region

		*Patient - Agent contrast*	
		Estimate	95% HPD
Human	Ambiguous	-0.0142	[-0.0553, 0.0268]
	Unambiguous	0.0232	[-0.0196, 0.0636]
Inanimate	Ambiguous	0.0197	[-0.0213, 0.0596]
	Unambiguous	-0.0037	[-0.0447, 0.0366]
		*Unambiguous – Ambiguous contrast*	
		Estimate	95% HPD
Human	Agent	0.0061	[-0.0350, 0.0464]
	Patient	0.0432	[0.0009, 0.0839]
Inanimate	Agent	0.0208	[-0.0199, 0.0608]
	Patient	-0.0027	[-0.0425, 0.0390]

For the second noun in the coordinated NP2 structure (NP2b), the reading times and estimates effects are shown in Fig. 5E-F and in Table S6. As in the Conjunction region, there is a reliable interaction of Animacy $\times$ Role. Resolving this interaction with the estimated marginal means in Table 8 as we did in the previous two regions suggests that agents were read faster in the animate condition but not in the inanimate conditions, as both the 95% HPDs in Table 8 for the animate conditions exclude 0 but the corresponding HPDs for the inanimates do not.

Thus, an interaction of Animacy $\times$ Semantic Role is consistent across all three words of the critical and spillover regions. In NP2a this manifests as the full three-way interaction with Ambiguity as well, while in the Conjunction and NP2b regions only Animacy and Semantic Role are involved. Moreover, across all three regions, the consistent pattern is that there greater RTs/reanalysis in the human conditions and that these effects are absent from the inanimate conditions.

Additionally, given the concerns about the impact of word length on other predictors raised by Experiment 1, we also fit models without word length for the two critical regions in Experiment 2 where word length varies (NP2a and NP2b). Unlike Experiment 1, neither the directionality nor the magnitude of the results changed in any substantial way (Tables S3 vs. S4 and Tables S6 vs. S7; see also Figs. 5B vs. 6 and Figs. 5F vs. 7). Thus, in this experiment, the effects favoring human over inanimate conditions and agent over patient conditions are consistent across model specifications. Furthermore, a model comparison between the models with and without word length showed a strong preference for the models with word length: the difference between models in a leave-one-out cross-validation comparison (approximated by importance sampling from the posterior) was $\Delta _{eldp} = 7.2$, SE $= 4.2$ for the NP2a models, and $\Delta _{eldp} = 6.8$, SE $= 4.2$ for the NP2b models. Thus, overall, the model comparison methods show strong preferences for the inclusion of word length as predictor, but also continue to support the other reported effects (unlike in Experiment 1).

**Table 7:** 95% HPD for the marginal means at the Conjunction region

		*Patient - Agent contrast*	
		Estimate	95% HPD
Human	Ambiguous	0.0522	[0.0208, 0.0843]
	Unambiguous	0.0315	[-0.0014, 0.0629]
Inanimate	Ambiguous	-0.0023	[-0.0337, 0.0288]
	Unambiguous	0.0079	[-0.0246, 0.0396]

### Discussion

The results of Experiment 2 show that there is a general tendency for faster reaction times when NP1 is an agent rather than a patient, i.e., the agent preference, which was found in the words that follow the disambiguating word, namely the conjunction and the coordinated noun. This re-confirms that the agent preference is a cross-linguistically robust processing principle (Haupt et al., 2008; Wang et al., 2009; Demiral et al., 2008; Bickel et al., 2015; Foley, 2020; Sauppe et al., 2023; Huber et al., 2023) and that it also persists in Basque (Erdocia et al., 2009).

**Table 8:** 95% HPD for the marginal means at the NP2b region.

		*Patient - Agent contrast*	
		Estimate	95% HPD
Human	Ambiguous	0.0435	[0.0065, 0.0804]
	Unambiguous	0.0380	[0.0002, 0.0749]
Inanimate	Ambiguous	0.0125	[-0.0240, 0.0498]
	Unambiguous	-0.0016	[-0.0372, 0.0362]

Additionally, in our experiments, ambiguous words are read faster than unambiguous words. This effect could be driven by frequency: ambiguous noun phrases have the ending*-ak*, which is the most frequent ending because it corresponds to*-ak* plural determiner and also to*-a-* singular determiner plus*-k* ergative case marker, and thus it can be used for singular agents, plural patients, sole arguments of intransitives marked with either absolutive (plural) or ergative (singular), and in copula sentences. Alternatively, a tendency for ambiguous conditions to be read faster, sometimes called the Ambiguity Advantage and found throughout the sentence comprehension literature, could be at work here (Swets et al., 2008). The reason behind this advantage for ambiguous input is still debated (Dillon et al., 2019; Sloggett et al., 2020), but it is well attested.

**Fig. 6:**
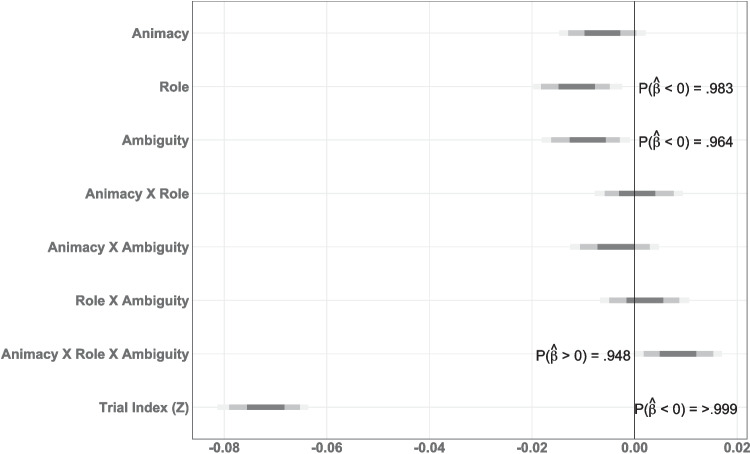
Posterior distributions by main effects of animacy, role, ambiguity, and their interactions for critical word (NP2a) in Experiment 2 (without word length as a predictor)

**Fig. 7:**
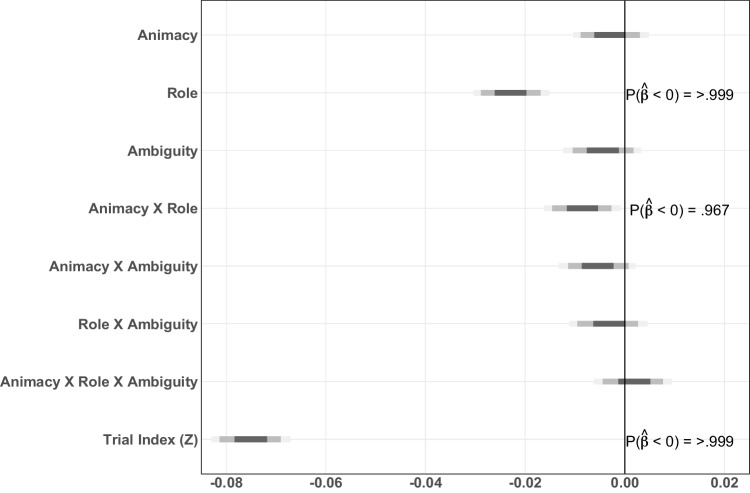
Posterior distributions by main effects of animacy, role, ambiguity, and their interactions for critical word +2 (NP2b) in Experiment 2 (without word length as a predictor)

More to the point of the present study, the results showed several differences in the way the agent preference applied to parsing when NP1 was animate vs. inanimate. In the disambiguating region at NP2a, this manifested as a three-way interaction, in which resolution preferences for ambiguous nouns showed that comprehenders preferred agent over patient readings, but only in the human-NP1 conditions, while inanimate-NP1 conditions showed either muted or no effect. In the conjunction and NP2b regions, a two-way interaction of Animacy $\times$ Role was found, which would be consistent if the parser had already resolved the ambiguity at NP2a, making the ambiguity factor irrelevant at this point in comprehension. This would be a fairly fast resolution of a reanalysis effect in self-paced reading, in which effects tend to be distributed over several spillover regions after the critical region (Mitchell, 1984). But a more consistent localization of sentence processing effects to the region of interest and not the extended spillover region has also been observed in some other languages, especially in languages with rich morphology like Basque (e.g., Foley, 2020) and also in other studies in Basque itself (Carreiras et al., 2010) and may be something that differs between languages more than previously thought. Whatever the reason for the disappearance of the three-way interaction, the Animacy $\times$ Role interaction shows that the parser does apply the agent preference to animate initial NPs (NP1), but does not apply it to inanimate NPs (either at all or at least much less).

These results are consistent with the idea that semantic features are able to influence when and how comprehenders rely on the agent preference, which makes them different from the syntactic features that had been tested in some previous studies. Specifically, animacy and the features that make it up impact how “good" an agent an NP can be fundamentally. By manipulating these features we can turn the agent preference on or off.

## General discussion

We have investigated the universality of the agent preference via two experiments that tested whether this processing bias holds in a context that, *a priori*, could have been biased against interpreting an unmarked initial NP as an agent in two ways: (i) by agents being overtly and distinctly marked by ergative case and (ii) by using inanimate nouns as agents. Across both experiments, we exploited a morphological overlap (syncretism) in the Basque case system that allowed us to simultaneously manipulate the animacy of NP1, its semantic role, and whether its role was ambiguous or unambiguous given the case marking. In Experiment 1, participants saw a NP-Verb-Auxiliary construction and the ambiguity at NP1 was resolved at the position of the auxiliary verb. This experiment used an argument-drop structure that is common in Basque discourse (Egurtzegi, 2023). We found suggestive differences between the conditions, but the results were confounded by word length. In Experiment 2, participants saw an NP-NP-Verb-Auxiliary construction where the role-ambiguity of NP1 was resolved by the unambiguous case marking on the second NP. Here, the results did show a reliable difference between processing agent- and patient-initial sentences, but it was strongly modulated by animacy. Only animate NPs showed a preference to be interpreted as agents. For inanimates, that is, in the absence of the prototypical agent features associated with animates, the agent preference was reduced or removed.

The current study echoes previous work in finding evidence for the agent preference. The ambiguous conditions in our experiments were read faster when they disambiguated toward an agent than toward a patient reading. These results replicated the conclusions of previous work by Erdocia et al. (2009), who also found an agent preference in Basque self-paced reading and ERP studies. Reanalysis effects have also been found when potential agents turned out to be patients in other languages with ergative case marking, such as Hindi and Georgian (Bickel et al., 2015; Foley, 2020), and in languages without ergative case (Wang et al., 2009; Demiral et al., 2008; Haupt et al., 2008).

Crucially, our work differed from that of Erdocia et al. (2009) in that we also included inanimate nouns in the agent role, showing that the agent preference applied to inanimate nouns much more weakly than to animate nouns. In other words, the agent preference can be robust to any number of factors in the grammar, such as the frequency of agent-initial constructions (Sauppe et al., 2023), or grammatical patterns such as ergativity that imply that nouns unmarked for case are most likely patients (Erdocia et al., 2009; Bickel et al., 2015), however, the same agent preference is not robust against variation in the intrinsic semantic features of referents that make them less good agents (Dowty, 1991; Silverstein, 1976; see also Sauppe et al., 2023), who found a patient preference for inanimate referents in the Oceanic language Äiwoo). Moreover, our findings are in line with Isasi-Isasmendi et al. (2023), who showed that intransitive subjects in Basque are preferentially interpreted as agents in the same way that transitive agents are, a finding which they argued cannot be captured based on strictly syntactic minimality principles. All of this is evidence that the agent preference is best characterized semantically as being about agents and not about subjects or similar syntactic notions.

The specific nature of Basque’s active-ergative alignment makes it a critical test language for comparing semantic role and syntactic accounts of the agent preference. Like Basque, both Georgian and Hindi have parts of the grammar that follow the ergative pattern and parts that do not. However, in those languages, the split is determined by aspect: in Hindi the perfective aspect has ergative alignment and the imperfective aspect has nominative-accusative alignment (Mahajan, 1990), while in Georgian the split is primarily aorist vs non-aorist (Harris, 1990; Foley, 2020). For Hindi, this means that although, as pointed out, the statistics of language bias unmarked nouns to be patients (Bickel et al., 2015) much the same as in Basque, the mechanism behind the bias is separate from the roles themselves. In contrast, in Basque, aspect is not a major divide in the case marking system; in fact, even surface-level aspect splits such as progressive have been argued not to be true exceptions to ergative alignment (Laka, 2006). Instead, Basque deviates from the classic ergative pattern by assigning absolutive case to only some intransitives; more agent-like intransitives tend to take ergative case instead. This pattern is frequently referred to as ’active’ or Split-S alignment (DeLancey, 1981; Harris, 1990; Rezac et al., 2014). This means that in Basque, more than in other languages with ergative case marking, ergative case is particularly closely tied to semantic agentivity (Aldai, 2009), rather than to the relative agentivity between two arguments or to grammatical function in a transitive clause (Laka, 2006, 2017).^8^ Although the agent/patient split in the intransitives does not fall perfectly along agent/patient lines, it is still the case that the outcome of this grammatical pattern will be that the link between morphological case and semantic role is tighter in Basque than it is in most other languages (Levin, 1983; De Rijk, 2007; Rezac et al., 2014). This makes it even more sensible for Basque comprehenders to rely on cues aligned with semantic agenthood, like animacy, to resolve case ambiguities in parsing.

The agent preference is of particular interest as a parsing bias because it is likely linked to a broader preference for agents in human cognition (Spelke & Kinzler, 2007). In eye tracking research, humans extract agent and patient information rapidly and spontaneously when visually inspecting events (Hafri et al., 2018) and pay more attention to agents than other elements of events, across ages (Cohn & Paczynski, 2013; Griffin & Bock, 2000; Galazka & Nyström, 2016; Sauppe & Flecken, 2021). Isasi-Isasmendi et al. (2023), for example, show that Basque and Spanish speakers preferentially encode visual information about agents in different tasks under time pressure (see Ünal et al., 2021, for additional evidence that agents are equally salient cross-linguistically). Hence, the agent preference as it applies to role-ambiguous arguments should probably be seen as part of a broader cognitive preference for agents.

An alternative account to explain the agent preference in sentence processing relates to minimality theories (Bornkessel-Schlesewsky & Schlesewsky, 2009; Bornkessel & Schlesewsky, 2006). The minimality principle states that online sentence processing is guided by a preference for local dependencies and smaller structures, a view inspired by independently established principles such as Minimal Attachment and Late Closure (Frazier & Rayner, 1982). According to the minimality account, the language parser only projects arguments and dependencies for which there is explicit evidence. For the processing of ambiguous initial noun phrases, the parser would first project an intransitive structure because this is the minimal structure and does not create any further dependency (as shown for Avar, Polinsky et al., 2012). When there is evidence against an intransitive interpretation (e.g., because a second NP is encountered), the parser would default to an agent-initial reading, as agents tend to be mapped to subjects in transitives.^9^ Hence, the agent preference might not necessarily be derived from a general cognitive preference for agents specifically, but from an underlying preference for more minimal sentence structures. Although in the current experiments we cannot disentangle the effects of the agent preference from those of minimality (or indeed, whether minimality could be seen as a source of the agent preference), further research should consider the processing differences between intransitive and transitive sentences in Basque.

## Conclusion

We have shown that the agent preference still holds for the least agent-processing-facilitating conditions, but this preference can be modulated. When looking at a language that systematically assigns ergative case to all agents across transitivity types, reading ambiguously marked inanimate agents is faster than reading their patient counterpart. However, processing inanimate agents is not as ideal as processing human agents. Additionally, further research should overall take languages with other alignment types than nominative into account when assessing how the language processing system functions.

## Supplementary Information

Below is the link to the electronic supplementary material.

Supplementary file 1 (pdf 35 KB)

## Data Availability

Data, and materials are available from https://osf.io/rkw4z/?view_only=fff9fb7b814747258a49ffbd7161bb35.

## Footnotes

1 In the sense of a transitive active subject or a transitive proto-agent role (Dowty, 1991; Bickel, 2010).

2 Argument-drop is frequent in Basque discourse (Egurtzegi, 2023).

3 The lexical verb may provide some information that could, together with the readers’ world knowledge, be used to disambiguate the ambiguous NP’s role. However, since each item contained only two different lexical verbs (Table 3), we refrained from modeling reading times in the verb region to avoid unidentifiable models.

4 The demographic and language background questionnaire can be found in the OSF repository under https://osf.io/vpe9g?view_only=fff9fb7b814747258a49ffbd7161bb35

5 See McElreath (2020) for justification of Bayesian thresholds.

6 Although note that this omission usually takes place when there is enough discourse information to retrieve the agent, which is not the case in an experiment like this.

7 Note that Foley (2020) and Sauppe et al. (2023) also find patient preferences, although with different distributions.

8 Georgian also has a split in the intransitive system, but the split along aspect still disrupts the mapping of ergative to agentivity.

9 That is, the subject of a transitive will be the argument with the highest degree of agentivity, relatively speaking, in active sentences (Dowty, 1991). In passives, of course, this relationship is broken, but passives are generally not considered minimal.

## Code Availability

Analysis scripts are likewise available from https://osf.io/rkw4z/?view_only=fff9fb7b814747258a49ffbd7161bb35.

## References

[CR1] Aldai, G. (2009). Is Basque morphologically ergative?: Western Basque vs. Eastern Basque. *Studies in Language. International Journal sponsored by the Foundation “Foundations of Language”*, *33*(4), 783–831,

[CR2] Altmann, G., & Steedman, M. (1988). Interaction with context during human sentence processing. *Cognition,**30*(3), 191–238. 3215002 10.1016/0010-0277(88)90020-0

[CR3] Bader, M., & Meng, M. (1999). Subject-object ambiguities in German embedded clauses: An across-the-board comparison. *Journal of Psycholinguistic Research,**28*(2), 121–143.

[CR4] Betancort, M., Carreiras, M., & Sturt, P. (2009). Short article: The processing of subject and object relative clauses in Spanish: An eye-tracking study. *Quarterly journal of experimental psychology,**62*(10), 1915–1929. 10.1080/17470210902866672 19424908

[CR5] Bickel, B. (2010). Grammatical relations typology. J.J. Song (Ed.), *The Oxford handbook of linguistic typology* (pp. 399–444). Oxford: Oxford University Press.

[CR6] Bickel, B., Witzlack-Makarevich, A., Choudhary, K. K., Schlesewsky, M., & Bornkessel-Schlesewsky, I. (2015). The neurophysiology of language processing shapes the evolution of grammar: Evidence from case marking. *PloS one,**10*(8), e0132819. 26267884 10.1371/journal.pone.0132819 PMC4534460

[CR7] Bornkessel, I., McElree, B., Schlesewsky, M., & Friederici, A. D. (2004). Multi-dimensional contributions to garden path strength: Dissociating phrase structure from case marking. *Journal of Memory and Language,**51*(4), 495–522.

[CR8] Bornkessel, I., & Schlesewsky, M. (2006). The extended argument dependency model: A neurocognitive approach to sentence comprehension across languages. *Psychological Review,**113*(4), 787–821. 10.1037/0033-295X.113.4.787 17014303 10.1037/0033-295X.113.4.787

[CR9] Bornkessel-Schlesewsky, I., & Schlesewsky, M. (2009). Minimality as vacuous distinctness: Evidence from cross-linguistic sentence comprehension. *Lingua,**119*(10), 1541–1559.

[CR10] Bornkessel-Schlesewsky, I., & Schlesewsky, M. (2009). The role of prominence information in the real-time comprehension of transitive constructions: a cross-linguistic approach. *Language and Linguistics Compass,**3*(1), 19–58.

[CR11] Bürkner, P.- C. (2017). Advanced Bayesian multilevel modeling with the R package brms. arXiv:1705.11123.

[CR12] Bürkner, P.- C., et al. (2017). brms: An R package for Bayesian multilevel models using Stan. *Journal of Statistical Software,**80*(1), 1–28.

[CR13] Carpenter, B., Gelman, A., Hoffman, M. D., Lee, D., Goodrich, B., Betancourt, M., & Riddell, A. (2017). Stan: A probabilistic programming language. *Journal of statistical software,**76*(1), 1–32. 10.18637/jss.v076.i01 PMC9788645 36568334

[CR14] Carreiras, M., Duñabeitia, J. A., Vergara, M., De La Cruz-Pavía, I., & Laka, I. (2010). Subject relative clauses are not universally easier to process: Evidence from Basque. *Cognition,**115*(1), 79–92. 20035934 10.1016/j.cognition.2009.11.012

[CR15] Choudhary, K.K. (2011). *Incremental argument interpretation in a split ergative language: Neurophysiological evidence from Hindi* (Unpublished doctoral dissertation). Max Planck Institute for Human Cognitive and Brain Sciences Leipzig.

[CR16] Clifton, C. (1992). Tracing the course of sentence comprehension: How lexical information is used. *Eye movements and visual cognition* (pp. 397–414). Springer.

[CR17] Clifton, C. (1993). Thematic roles in sentence parsing. *Canadian Journal of Experimental Psychology/Revue canadienne de psychologie expérimentale,**47*(2), 222. 8364531 10.1037/h0078817

[CR18] Cohn, N., & Paczynski, M. (2013). Prediction, events, and the advantage of Agents: The processing of semantic roles in visual narrative. *Cognitive Psychology,**67*(3), 73–97. 10.1016/j.cogpsych.2013.07.002 23959023 10.1016/j.cogpsych.2013.07.002 PMC3895484

[CR19] Comrie, B. (1989). *Language universals and linguistic typology: Syntax and morphology*. University of Chicago press.

[CR20] DeLancey, S. (1981). An interpretation of split ergativity and related patterns. *Language,**57*, 626–657.

[CR21] Demiral, ŞB., Schlesewsky, M., & Bornkessel-Schlesewsky, I. (2008). On the universality of language comprehension strategies: Evidence from Turkish. *Cognition,**106*(1), 484–500. 17336956 10.1016/j.cognition.2007.01.008

[CR22] De Rijk, R. (2007). *Standard Basque: A progressive grammar*. Cambridge, MA: MIT Press. (Includes bibliographical references and index)

[CR23] Deutsch, A., & Bentin, S. (2001). Syntactic and semantic factors in processing gender agreement in Hebrew: Evidence from ERPs and eye movements. *Journal of Memory and Language,**45*(2), 200–224.

[CR24] Dillon, B., Andrews, C., Rotello, C. M., & Wagers, M. (2019). A new argument for co-active parses during language comprehension. *Journal of Experimental Psychology: Learning, Memory, and Cognition,**45*(7), 1271. 30124311 10.1037/xlm0000649

[CR25] Dowty, D. (1991). *Thematic proto-roles and argument selection. language,**67*(3), 547–619.

[CR26] Dröge, A., Rabs, E., Fleischer, J., Billion, S. K., Meyer, M., Schmid, S., & Bornkessel-Schlesewsky, I. (2020). Case syncretism, animacy, and word order in Continental West Germanic: Neurolinguistic evidence from a comparative study on Standard German, Zurich German, and Fering (North Frisian). *Journal of Germanic Linguistics,**32*(3), 217–310.

[CR27] Drummond, A. (2020). *Ibex Farm* (Vol. 831).

[CR28] Egurtzegi, A. (2023). *Ergativity in language production and comprehension* (Doctoral dissertation, University of Zürich”).10.5167/uzh-237659

[CR29] Erdocia, K., Laka, I., Mestres-Missé, A., & Rodriguez-Fornells, A. (2009). Syntactic complexity and ambiguity resolution in a free word order language: Behavioral and electrophysiological evidences from Basque. *Brain and Language,**109*(1), 1–17. 19223065 10.1016/j.bandl.2008.12.003

[CR30] Ferreira, F., & Clifton, C., Jr. (1986). The independence of syntactic processing. *Journal of Memory and Language,**25*(3), 348–368.

[CR31] Foley, S. (2020). *Case, agreement, and sentence processing in Georgian (Unpublished doctoral dissertation)*. Santa Cruz: University of California.

[CR32] Frazier, L., & d’Arcais, G. B. F. (1989). Filler driven parsing: A study of gap filling in Dutch. *Journal of Memory and Language,**28*(3), 331–344.

[CR33] Frazier, L., & Rayner, K. (1982). Making and correcting errors during sentence comprehension: Eye movements in the analysis of structurally ambiguous sentences. *Cognitive Psychology,**14*(2), 178–210. 10.1016/0010-0285(82)90008-1

[CR34] Friederici, A. D., Mecklinger, A., Spencer, K. M., Steinhauer, K., & Donchin, E. (2001). Syntactic parsing preferences and their on-line revisions: A spatio-temporal analysis of event-related brain potentials. *Cognitive Brain Research,**11*(2), 305–323. 11275491 10.1016/s0926-6410(00)00065-3

[CR35] Galazka, M., & Nyström, P. (2016). Infants’ preference for individual agents within chasing interactions. *Journal of Experimental Child Psychology,**147*, 53–70. 10.1016/j.jecp.2016.02.010 27017143 10.1016/j.jecp.2016.02.010

[CR36] Gennari, S. P., & MacDonald, M. C. (2008). Semantic indeterminacy in object relative clauses. *Journal of Memory and Language,**58*(2), 161–187. 19724662 10.1016/j.jml.2007.07.004 PMC2735264

[CR37] Griffin, Z. M., & Bock, K. (2000). What the eyes say about speaking. *Psychological Science,**11*(4), 274–279. 11273384 10.1111/1467-9280.00255 PMC5536117

[CR38] Hafri, A., Trueswell, J. C., & Strickland, B. (2018). Encoding of event roles from visual scenes is rapid, spontaneous, and interacts with higher-level visual processing. *Cognition,**175*, 36–52. 29459238 10.1016/j.cognition.2018.02.011 PMC5879027

[CR39] Harris, A. C. (1990). Georgian: A language with active case marking: A reply to BG Hewitt. *Lingua,**80*(1), 35–53.

[CR40] Haupt, F. S., Schlesewsky, M., Roehm, D., Friederici, A. D., & Bornkessel-Schlesewsky, I. (2008). The status of subject-object reanalyses in the language comprehension architecture. *Journal of Memory and Language,**59*(1), 54–96.

[CR41] Hemforth, B., Konieczny, L., Strube, G. (1993). Incremental syntax processing and parsing strategies. *Proceedings of the 15th annual conference of the cognitive science society* (pp. 539–545).

[CR42] Huber, E., Sauppe, S., Isasi-Isasmendi, A., Bornkessel-Schlesewsky, I., Merlo, P., & Bickel, B. (2023). Surprisal from language models can predict ERPs in processing predicate-argument structures only if enriched by an Agent Preference principle. *Neurobiology of Language*. 10.1162/nol_a_00121 10.1162/nol_a_00121 PMC11025647 38645615

[CR43] Isasi-Isasmendi, A., Andrews, C., Flecken, M., Laka, I., Daum, M. M., Meyer, M., & Sauppe, S. (2023). The agent preference in visual event apprehension. *Open Mind,**7*, 240–282. 10.1162/opmi_a_00083 37416075 10.1162/opmi_a_00083 PMC10320828

[CR44] Isasi-Isasmendi, A., Sauppe, S., Andrews, C., Laka, I., Meyer, M., & Bickel, B. (2023). Incremental sentence processing is guided by a preference for agents: EEG evidence from Basque. *Language, Cognition and Neuroscience,**39*(1), 76–97. 10.1080/23273798.2023.2250023

[CR45] Jegerski, J. (2013). Self-paced reading. J. Jegerski and B. VanPatten (Eds.), *Research methods in second language psycholinguistics* (pp. 36–65). Routledge.

[CR46] Kretzschmar, F., Bornkessel-Schlesewsky, I., Staub, A., Roehm, D., Schlesewsky, M. (2012). Prominence facilitates ambiguity resolution: On the interaction between referentiality, thematic roles and word order in syntactic reanalysis. M. Lamers and P. De Swart (Eds.), *Case, Word Order and Prominence* (pp. 239–271). Springer.

[CR47] Laka, I. (2006). Deriving split ergativity in the progressive: The case of Basque. A. Johns, D. Massam, and J. Ndayiragije (Eds.), *Ergativity: Emerging issues* (pp. 173–195). Springer.

[CR48] Laka, I. (2017). Ergative need not split: An exploration into the TotalErg Hypothesis. J. Coon, D. Massam, and L. Travis (Eds.), *The Oxford Handbook of Ergativity* (pp. 159–174). Oxford University Press Oxford.

[CR49] Lenth, R.V. (2023). emmeans: Estimated marginal means, aka least-squares means [Computer software manual]. Retrieved from https://CRAN.R-project.org/package=emmeans (R package version 1.8.4-1)

[CR50] Levin, B. (1983). *On the nature of ergativity*. Cambridge, MA: MIT dissertation.

[CR51] Lowder, M. W., & Gordon, P. C. (2012). The pistol that injured the cowboy: Difficulty with inanimate subject-verb integration is reduced by structural separation. *Journal of Memory and Language,**66*(4), 819–832.

[CR52] Mahajan, A.K. (1990). *The A/A-bar distinction and movement theory* (Unpublished doctoral dissertation). Massachusetts Institute of Technology.

[CR53] Mak, W. M., Vonk, W., & Schriefers, H. (2006). Animacy in processing relative clauses: The hikers that rocks crush. *Journal of Memory and Language,**54*(4), 466–490.

[CR54] Manterola, J. (2008). Is Basque an agglutinative language? *Basque studies symposium.*

[CR55] Marslen-Wilson, W. (1973). Linguistic structure and speech shadowing at very short latencies. *Nature,**244*(5417), 522–523. 4621131 10.1038/244522a0

[CR56] McElreath, R. (2020). *Statistical rethinking: A Bayesian course with examples in R and Stan*. CRC press.

[CR57] Mitchell, D. (1984). An evaluation of subject-paced reading tasks and other methods of investigating immediate processes in reading. D.E. Kieras and M.A. Just (Eds.), *New methods in in reading comprehension research.* Erlbaum, Hillsdale, N.J.

[CR58] Paape, D., & Vasishth, S. (2016). Local coherence and preemptive digging-in effects in German. *Language and Speech,**59*(3), 387–403. 29924534 10.1177/0023830915608410

[CR59] Payne, B., & Stine-Morrow, E. (2014). Adult age differences in wrap-up during sentence comprehension: Evidence from ex-Gaussian distributional analyses of reading time. *Psychology and Aging,**29*(2), 213. 24955990 10.1037/a0036282

[CR60] Polinsky, M., Gallo, C. G., Graff, P., & Kravtchenko, E. (2012). Subject preference and ergativity. *Lingua,**122*(3), 267–277.

[CR61] R Core Team (2023). R: A language and environment for statistical computing [Computer software manual]. Vienna. Retrieved from http://www.R-project.org

[CR62] Rezac, M., Albizu, P., & Etxepare, R. (2014). The structural ergative of Basque and the theory of Case. *Natural Language & Linguistic Theory,**32*, 1273–1330.

[CR63] Sassenhagen, J., & Alday, P. M. (2016). A common misapplication of statistical inference: Nuisance control with null-hypothesis significance tests. *Brain and Language,**162*, 42–45. 27543688 10.1016/j.bandl.2016.08.001

[CR64] Sauppe, S., & Flecken, M. (2021). Speaking for seeing: Sentence structure guides visual event apprehension. *Cognition,**206*, 104516. 10.1016/j.cognition.2020.104516 10.1016/j.cognition.2020.104516 33228969

[CR65] Sauppe, S., Næss, Å., Roversi, G., Meyer, M., Bornkessel-Schlesewsky, I., & Bickel, B. (2023). An agent-first preference in a patient-first language during sentence comprehension. *Cognitive Science,**47*(9), e13340. 10.1111/cogs.13340 37715510 10.1111/cogs.13340

[CR66] Schielzeth, H. (2010). Simple means to improve the interpretability of regression coefficients. *Methods in Ecology and Evolution,**1*(2), 103–113.

[CR67] Schlesewsky, M., Fanselow, G., Kliegl, R., Krems, J. (2000). The subject preference in the processing of locally ambiguous Wh-questions in German. B. Hemforth and L. Konieczny (Eds.), *German Sentence Processing* (pp. 65–93). Springer.

[CR68] Silverstein, M. (1976). Hierarchy of features and ergativity. R.M.W. Dixon (Ed.), *Grammatical categories in Australian languages* (p.112-171). New Jersey: Humanities Press.

[CR69] Sloggett, S., Van Handel, N., Sasaki, K., Duff, J., Rich, S., Orth, W.. Rysling, A. (2020). “Ambiguous” isn’t “underspecified”: Evidence from the Maze task. *Poster presented at the 33rd annual CUNY Conference on human sentence processing, University of Massachusetts, Amherst.*

[CR70] Spelke, E. S., & Kinzler, K. D. (2007). Core knowledge. *Developmental Science,**10*(1), 89–9. 10.1111/j.1467-7687.2007.00569.x 17181705 10.1111/j.1467-7687.2007.00569.x

[CR71] Swets, B., Desmet, T., Clifton, C., & Ferreira, F. (2008). Underspecification of syntactic ambiguities: Evidence from self-paced reading. *Memory & Cognition,**36*(1), 201–216. 10.3758/mc.36.1.201 18323075

[CR72] Traxler, M. J., Williams, R. S., Blozis, S. A., & Morris, R. K. (2005). Working memory, animacy, and verb class in the processing of relative clauses. *Journal of Memory and Language,**53*(2), 204–224.

[CR73] Trueswell, J. C., Tanenhaus, M. K., & Garnsey, S. M. (1994). Semantic influences on parsing: Use of thematic role information in syntactic ambiguity resolution. *Journal of Memory and Language,**33*(3), 285–318.

[CR74] Tunmer, W.E. (1985). The acquisition of the sentient-nonsentient distinction and its relationship to causal reasoning and social cognition. *Child development*. 989–1000,

[CR75] Ünal, E., Richards, C., Trueswell, J. C., & Papafragou, A. (2021). Representing agents, patients, goals and instruments in causative events: A cross-linguistic investigation of early language and cognition. *Developmental Science,**24*(6), e13116. 10.1111/desc.13116 33955664

[CR76] Wang, L., Schlesewsky, M., Bickel, B., & Bornkessel-Schlesewsky, I. (2009). Exploring the nature of the ‘subject’-preference: evidence from the online comprehension of simple sentences in Mandarin Chinese. *Language and Cognitive Processes,**24*(7–8), 1180–1226.

[CR77] Yetano, I., Duñabeitia, J.A., Laka, I. (2019). Processing preferences in an ergative language: Evidence from Basque postnominal relative clauses. I. Laka (Ed.), *Hitzak sarean. pello salabururi esker onez* (pp. 137–150).

[CR78] Zuur, A. F., Ieno, E. N., & Elphick, C. S. (2010). A protocol for data exploration to avoid common statistical problems. *Methods in Ecology and Evolution,**1*(1), 3–14. 10.1111/j.2041-210X.2009.00001.x

