# Supplementary material for: The effect of animacy on the agent preference: Self-paced reading evidence from Basque
Source: Mem Cognit. 2025 Apr 30;53(7):2056–75. doi: 10.3758/s13421-025-01698-w (PMC12589303; doi:10.3758/s13421-025-01698-w)
Supplement: Supplementary file 1 — (pdf 35 KB) [file 13421_2025_1698_MOESM1_ESM.pdf]

Supplementary Materials to Egurtzegi, A., Sauppe, S., Isasi-Isasmendi Landaluze, A., Martínez de la Hidalga, G., Schlesewsky, M., Bornkessel-Schlesewsky, I., Laka, I., Meyer, M., Bickel, B., and Andrews, C. *The effect of animacy on the agent preference: Self-paced reading evidence from Basque*

S.1 Model summaries

Table S1: Posterior parameter estimates from Bayesian hierarchical regression predicting reading times for the critical word (AUX) in Experiment 1 (with word length as a predictor). We report $P(\hat{\beta} > 0)$ when mean $\hat{\beta} > 0$ and $P(\hat{\beta} < 0)$ when mean $\hat{\beta} < 0$. Highlighted terms have a $P(> | < 0) >= 0.9$.

Parameter	Mean $\hat{\beta}$	SE	Highest Density Interval Quantiles 5%	95%	$P(\hat{\beta} < \| > 0)$	Variance Inflation Factor
Intercept	6.16	0.0614	6.1	6.22		
Animacy (Human)	0.00920	0.0105	0	0.02	P(> 0) = 0.957	1.00
Role (Agent)	0.0271	0.0396	-0.01	0.07	P(> 0) = 0.910	14.50
Ambiguity (Ambiguous)	-0.0143	0.0218	-0.04	0.01	P(< 0) = 0.902	4.38
Animacy × Role	-0.00605	0.0104	-0.02	0	P(< 0) = 0.873	1.00
Animacy × Ambiguity	-0.00613	0.0105	-0.02	0	P(< 0) = 0.872	1.00
Role × Ambiguity	0.0252	0.0401	-0.02	0.06	P(> 0) = 0.892	14.52
Animacy × Role × Ambiguity	-0.00258	0.0104	-0.01	0.01	P(< 0) = 0.687	1.00
Word Length (standardized)	0.0632	0.0601	0	0.12	P(> 0) = 0.979	31.88
Trial Index (standardized)	-0.0734	0.0107	-0.08	-0.06	P(< 0) > 0.999	1.00
σ	0.223	0.00761	0.22	0.23		

Table S2: Posterior parameter estimates from Bayesian hierarchical regression predicting reading times for the critical word (AUX) in Experiment 1 (without word length as a predictor). We report $P(\hat{\beta} > 0)$ when mean $\hat{\beta} > 0$ and $P(\hat{\beta} < 0)$ when mean $\hat{\beta} < 0$. Highlighted terms have a $P(> | < 0) >= 0.9$.

Parameter	Mean $\hat{\beta}$	SE	Highest Density Interval Quantiles 5%	95%	$P(\hat{\beta} < \| > 0)$	Variance Inflation Factor
Intercept	6.16	0.0614	6.1	6.22		
Animacy (Human)	0.00918	0.0104	0	0.02	P(> 0) = 0.956	1.00
Role (Agent)	-0.0131	0.0105	-0.02	0	P(< 0) = 0.993	1.00
Ambiguity (Ambiguous)	0.00591	0.0103	0	0.02	P(> 0) = 0.868	1.00
Animacy × Role	-0.00580	0.0105	-0.02	0	P(< 0) = 0.862	1.00
Animacy × Ambiguity	-0.00629	0.0104	-0.02	0	P(< 0) = 0.880	1.00
Role × Ambiguity	-0.0154	0.0105	-0.03	0	P(< 0) = 0.998	1.00
Animacy × Role × Ambiguity	-0.00247	0.0103	-0.01	0.01	P(< 0) = 0.683	1.00
Trial Index (standardized)	-0.0729	0.0106	-0.08	-0.06	P(< 0) > 0.999	1.00
σ	0.223	0.00756	0.22	0.23		

Table S3: Posterior parameter estimates from Bayesian hierarchical regression predicting reading times for the critical word (NP2a) in Experiment 2 (with word length as a predictor). The posterior probability of a parameter is reported when it is at least 90%. We report $P(\hat{\beta} > 0)$ when mean $\hat{\beta} > 0$ and $P(\hat{\beta} < 0)$ when mean $\hat{\beta} < 0$. Highlighted terms have a $P(> | < 0) >= 0.9$.

Parameter	Mean $\hat{\beta}$	SE	Highest Density Interval Quantiles 5%	95%	$P(\hat{\beta} < \| > 0)$	Variance Inflation Factor
Intercept	6.20	0.0652	6.14	6.27		
Animacy	-0.00615	0.0102	-0.02	0	P(< 0) = 0.884	1.00
Role	-0.00312	0.0105	-0.01	0.01	P(< 0) = 0.722	1.06
Ambiguity	-0.00843	0.0101	-0.02	0	P(< 0) = 0.951	1.00
Animacy × Role	0.000918	0.0102	-0.01	0.01	P(> 0) = 0.569	1.00
Animacy × Ambiguity	-0.00391	0.0104	-0.01	0.01	P(< 0) = 0.769	1.00
Role × Ambiguity	0.00172	0.0101	-0.01	0.01	P(> 0) = 0.634	1.00
Animacy × Role × Ambiguity	0.00760	0.0102	0	0.02	P(> 0) = 0.927	1.00
Word Length (standardized)	0.0382	0.0120	0.03	0.05	P(> 0) > 0.999	1.06
Trial Index (standardized)	-0.0720	0.0104	-0.08	-0.06	P(< 0) > 0.999	1.00
σ	0.222	0.00740	0.21	0.23		

Table S4: Posterior parameter estimates from Bayesian hierarchical regression predicting reading times for the critical word (NP2a) in Experiment 2 (without word length as a predictor). The posterior probability of a parameter is reported when it is at least 90%. We report $P(\hat{\beta} > 0)$ when mean $\hat{\beta} > 0$ and $P(\hat{\beta} < 0)$ when mean $\hat{\beta} < 0$. Highlighted terms have a $P(> | < 0) >= 0.9$.

Parameter	Mean $\hat{\beta}$	SE	Highest Density Interval Quantiles 5%	95%	$P(\hat{\beta} < \| > 0)$	Variance Inflation Factor
Intercept	6.20	0.0679	6.14	6.27		
Animacy	-0.00627	0.0101	-0.02	0	P(< 0) = 0.889	1.00
Role	-0.0112	0.0103	-0.02	0	P(< 0) = 0.983	1.00
Ambiguity	-0.00936	0.0102	-0.02	0	P(< 0) = 0.964	1.00
Animacy × Role	0.000591	0.0102	-0.01	0.01	P(> 0) = 0.544	1.00
Animacy × Ambiguity	-0.00373	0.0103	-0.01	0.01	P(< 0) = 0.764	1.00
Role × Ambiguity	0.00193	0.0104	-0.01	0.01	P(> 0) = 0.643	1.00
Animacy × Role × Ambiguity	0.00849	0.0102	0	0.02	P(> 0) = 0.948	1.00
Trial Index (standardized)	-0.0721	0.0105	-0.08	-0.06	P(< 0) > 0.999	1.00
σ	0.221	0.00738	0.21	0.23		

Table S5: Posterior parameter estimates from Bayesian hierarchical regression predicting reading times for the critical word +1 (Conjunction) in Experiment 2 (word length does not vary between conditions in this region and therefore is not included as a predictor). The posterior probability of a parameter is reported when it is at least 90%. We report $P(\hat{\beta} > 0)$ when mean $\hat{\beta} > 0$ and $P(\hat{\beta} < 0)$ when mean $\hat{\beta} < 0$. Highlighted terms have a $P(> \mid < 0) >= 0.9$.

Parameter	Mean $\hat{\beta}$	SE	Highest Density Interval Quantiles 5%	95%	$P(\hat{\beta} < \mid > 0)$	Variance Inflation Factor
Intercept	6.12	0.0520	6.07	6.17		
Animacy	-0.00255	0.00796	-0.01	0.01	P(< 0) = 0.734	1.00
Role	-0.0112	0.00804	-0.02	0	P(< 0) = 0.997	1.00
Ambiguity	-0.00189	0.00789	-0.01	0.01	P(< 0) = 0.679	1.00
Animacy × Role	-0.00977	0.00799	-0.02	0	P(< 0) = 0.991	1.00
Animacy × Ambiguity	-0.0045	0.00797	-0.01	0	P(< 0) = 0.866	1.00
Role × Ambiguity	-0.00127	0.00800	-0.01	0.01	P(< 0) = 0.625	1.00
Animacy × Role × Ambiguity	-0.00391	0.00795	-0.01	0	P(< 0) = 0.829	1.00
Trial Index (standardized)	-0.0533	0.00815	-0.06	-0.05	P(< 0) > 0.999	1.00
σ	0.173	0.00575	0.17	0.18		

Table S6: Posterior parameter estimates from Bayesian hierarchical regression predicting reading times for the critical word +2 (NP2-2) in Experiment 2 (with word length as a predictor). The posterior probability of a parameter is reported when it is at least 90%. We report $P(\hat{\beta} > 0)$ when mean $\hat{\beta} > 0$ and $P(\hat{\beta} < 0)$ when mean $\hat{\beta} < 0$. Highlighted terms have a $P(> \mid < 0) >= 0.9$.

Parameter	Mean $\hat{\beta}$	SE	Highest Density Interval Quantiles 5%	95%	$P(\hat{\beta} < \mid > 0)$	Variance Inflation Factor
Intercept	6.11	0.0647	6.05	6.18		
Animacy	-0.00252	0.00905	-0.01	0.01	P(< 0) = 0.704	1.00
Role	-0.0116	0.00957	-0.02	0	P(< 0) = 0.991	1.09
Ambiguity	-0.00464	0.00916	-0.01	0	P(< 0) = 0.840	1.00
Animacy × Role	-0.00879	0.00900	-0.02	0	P(< 0) = 0.971	1.00
Animacy × Ambiguity	-0.00502	0.00915	-0.01	0	P(< 0) = 0.860	1.00
Role × Ambiguity	-0.00245	0.00914	-0.01	0.01	P(< 0) = 0.701	1.01
Animacy × Role × Ambiguity	0.00105	0.00919	-0.01	0.01	P(> 0) = 0.587	1.00
Word Length (standardized)	0.0530	0.0127	0.04	0.07	P(> 0) > 0.999	1.09
Trial Index (standardized)	-0.0749	0.00932	-0.08	-0.07	P(< 0) > 0.999	1.00
σ	0.198	0.00666	0.19	0.2		

Table S7: Posterior parameter estimates from Bayesian hierarchical regression predicting reading times for the critical word +2 (NP2-2) in Experiment 2 (without word length as a predictor). The posterior probability of a parameter is reported when it is at least 90%. We report $P(\hat{\beta} > 0)$ when mean $\hat{\beta} > 0$ and $P(\hat{\beta} < 0)$ when mean $\hat{\beta} < 0$. Highlighted terms have a $P(> | < 0) >= 0.9$.

Parameter	Mean $\hat{\beta}$	SE	Highest Density Interval Quantiles 5%	95%	$P(\hat{\beta} < \| > 0)$	Variance Inflation Factor
Intercept	6.11	0.0647	6.05	6.18		
Animacy	-0.00296	0.00908	-0.01	0.01	P(< 0) = 0.738	1.00
Role	-0.0229	0.00913	-0.03	-0.01	P(< 0) > 0.999	1.00
Ambiguity	-0.00445	0.00931	-0.01	0	P(< 0) = 0.826	1.00
Animacy × Role	-0.00855	0.00908	-0.02	0	P(< 0) = 0.967	1.00
Animacy × Ambiguity	-0.00538	0.00920	-0.01	0	P(< 0) = 0.873	1.00
Role × Ambiguity	-0.00325	0.00934	-0.01	0.01	P(< 0) = 0.757	1.00
Animacy × Role × Ambiguity	0.00170	0.00928	-0.01	0.01	P(> 0) = 0.640	1.00
Trial Index (standardized)	-0.0751	0.00940	-0.08	-0.07	P(< 0) > 0.999	1.01
σ	0.198	0.00656	0.19	0.2		
